# Increased Carcinogenic Action of Dimethylnitrosamine after Prior Administaion of Carbon Tetrachloride

**DOI:** 10.1038/bjc.1973.57

**Published:** 1973-06

**Authors:** A. W. Pound, T. A. Lawson, Lorraine Horn

## Abstract

**Images:**


*Br. J. Cancer* (1973) **27**, 451

### INCREASED CARCINOGENIC ACTION OF DIMETHYLNITROSAMINE AFTER PRIOR ADMINISTRATION OF CARBON TETRACHLORIDE

A. W. POUND, T. A. LAWSON AND LORRAINE HORN

*From the Department of Pathology, University of Queensland, Brisbane, Australia*

Received 21 October 1972. Accepted 20 February 1973

**Summary.—Rats were given a single dose of dimethylnitrosamine (DMN, 20 mg/kg body weight) alone or 42 or 60 hours after a non-lethal hepatotoxic dose of carbon tetrachloride ($CCl_4$) and killed 12 months later. DMN alone produced no tumours in the kidney and a few in the liver, but when given 42 hours after $CCl_4$, tumours formed in the kidneys and the number in the liver was increased. When given after 60 hours, the incidence of kidney tumours was less but that of liver tumours was further increased. A larger dose of DMN (40 mg/kg) was tolerated 42 hours after $CCl_4$ and enhanced the number of kidney and liver tumours, the latter apparently due to an increased proportion of cholangiomata. Numerous small focal proliferations of atypical liver cells and of bile duct epithelium were observed after treatment with DMN. The incidence of these lesions in the different experimental treatments varied in a similar manner to the liver tumours.**

MANY nitroso compounds are acutely toxic and potently carcinogenic for many organs and tissues (Magee and Barnes, 1967). Dimethylnitrosamine (DMN) produced haemorrhagic necrosis in the liver, haemorrhagic lesions in the lung, haemorrhagic ascites and pleural effusions in a number of animal species (Barnes and Magee, 1954). On prolonged feeding to animals, it produced tumours of the liver, kidneys and other organs (Magee and Barnes, 1962).

The toxic effects were reduced in animals given a protein-free diet (McLean and Verschuuren, 1969) or treated by other means that reduced the formation of microsomal enzymes concerned in the metabolism of DMN (Venkatesan, Arcos and Argus, 1968; Venkatesan, Argus and Arcos, 1970; Fiume *et al.*, 1970; Schmähl *et al.*, 1971; Magour and Nievel, 1971; Swann and McLean, 1971; Mirvish and Sidransky, 1971; Somogyi *et al.*, 1972; Pound, Horn and Lawson, 1973). On the other hand, a protein-free diet increased the susceptibility of animals to the carcinogenic action of DMN for the kidneys but not apparently for the liver (McLean and Magee, 1970).

The hypothesis that proliferating cells are more susceptible to the action of a variety of chemical carcinogens, was based on the correlation of the number of tumours initiated by urethane in proliferating epidermis with the number of cells in DNA synthesis, and was supported by an increased number of tumours of the liver in mice given urethane or dimethylbenzanthracene after partial hepatectomy (Pound, 1968). A similar increase after partial hepatectomy has been reported in mice given urethane (Chernozemski and Warwick, 1970) and in rats given DMN (Craddock, 1971), and related to the period of DNA synthesis.

The possibility was raised that the tumour yield in the liver would be increased if DMN were given in the period of regeneration that followed a necrotizing dose of a hepatotoxin that itself was not carcinogenic. Carbon tetrachloride ($CCl_4$) produced centrilobular coagulative necrosis in the liver followed by regeneration (Cameron and Karunaratne, 1936; Hoffman *et al.*, 1955). DNA synthesis in the remaining liver commenced within 15 hours, followed after a short interval by active mitosis (Leevy *et al.*, 1959). The carcinogenicity of $CCl_4$ for mice is manifested only in certain susceptible strains after prolonged and frequent dosage, and in rats even this weak carcinogenic action is controversial (Clayson, 1962).

This paper records the occurrence of tumours of the liver and kidney induced in rats by a single dose of DMN given a short interval after a single necrosis-producing dose of $CCl_4$.

### MATERIALS AND METHODS

*Animals.*—Random bred male Sprague–Dawley rats were maintained on standard rat pellets manufactured to a formula supplied by the Queensland Institute for Medical Research by Bunge (Australia) Pty Ltd., Warwick. It contained approximately 20% protein, 4·4% fat, 60% carbohydrate and fibre, 10% moisture with an added mineral and vitamin supplement. Water was supplied *ad libitum*. The rats were 12–16 weeks of age and 260–360 g weight at the beginning of each experiment.

*Chemicals.*—Carbon tetrachloride, A.R., was obtained from British Drug Houses Ltd, Poole, Great Britain. Dimethylnitrosamine from K. & K. Laboratories, New York, was redistilled in the laboratory. $CCl_4$ was administered by stomach tube, under light ether anaesthesia, as 1·5 ml of a solution in peanut oil. DMN was administered as an intraperitoneal injection in 1 ml of saline.

*Histological methods.*—Tissues for histological examination were fixed in 4% formaldehyde in buffered saline. The material was processed, sections cut and stained by routine methods.

*Experimental.*—Rats were treated with $CCl_4$ (2·5 ml/kg). Group 1, 60 rats, received no further treatment. Group 2, 32 rats, were given DMN (20 mg/kg) 42 hours after the dose of $CCl_4$, and Group 3, 35 rats, received DMN (20 mg/kg) 60 hours after the $CCl_4$. As control groups, 33 rats were set aside with no treatment. Two groups of 18 rats were given $CCl_4$ (2·5 ml/kg) followed by DMN (40 mg/kg) 42 and 60 hours later respectively. All rats in the latter group died within 6 days.

The animals were kept for 12 months, the survivors killed and subjected to autopsy. The liver and kidneys were examined with the naked eye for the presence of tumours. A full longitudinal section of each kidney and representative full sections of the lobes of the liver were examined microscopically. Occasionally sections were taken of other tissues. The few (3–5) rats in each group that died were ignored, as the deaths appeared to be due to random factors.

### RESULTS

### *Control animals*

None of the 55 surviving rats treated with $CCl_4$ alone, nor of the 33 rats that had had no treatment, had any lesions macroscopically or microscopically in the liver or kidneys. The animals showed bronchiectasis of varying degrees, with evidence of chronic infection that is endemic in this colony. This did not vary between the treatment groups.

### *Animals treated with DMN*

Animals treated with DMN alone or DMN after $CCl_4$ showed the usual bronchiectatic changes; no tumours were seen in the lungs. A proportion of the animals (Table I) showed tumours or tumour-like lesions in the liver and kidneys. No other histological differences were observed in these organs between the groups of animals; in particular there was no evidence of cirrhosis, chronic liver disease or chronic kidney disease. Since there appeared to be a correlation between the incidence of the different lesions they were classified as follows.

### *Lesions in the Liver*

*Hepatocellular tumours* were characteristic masses of recognizable liver cells growing in an unco-ordinated fashion, *i.e.* not presenting the usual arrangement of liver cells in plates separated by sinusoids, which showed evidence of expansive growth manifested by the thrusting aside of the surrounding liver cells and growth

TABLE I.—*Number of Tumours and Tumour-like Lesions Found in Rats Treated with $CCl_4$, DMN, or $CCl_4$ Followed by DMN*

| | | | DMN 20 mg/kg | | | | | | DMN 40 mg/kg 42 hours after $CCl_4$ | |
|---|---|---|---|---|---|---|---|---|---|---|
| | | | DMN only | | 42 hours after $CCl_4$ | | 60 hours after $CCl_4$ | | | |
| | | $CCl_4$ only | Lesions | Animals | Lesions | Animals | Lesions | Animals | Lesions | Animals |
| Liver | Hepatocellular tumours | 0 | 2 | 2 | 3 | 3 | 10 | 7 | 5 | 4 |
| | Focal proliferations | 0 | 4 | 3 | 14 | 9 | 26 | 12 | 28 | 12 |
| | Small focal proliferations | 0 | 3 | 3 | 7 | 6 | 8 | 8 | 16 | 11 |
| | Cholangiomata | 0 | 0 | 0 | 0 | 0 | 4 | 3 | 6 | 6 |
| | Duct proliferations | 0 | 0 | 0 | 3 | 3 | 6 | 5 | 20 | 12 |
| | Cysts | 0 | 1 | 1 | 1 | 1 | 2 | 2 | 23 | 11 |
| Kidney | Papillary adenocarcinoma | 0 | 0 | 0 | 4 | 4 | 1 | 1 | 17 | 10 |
| | Nephroblastoma | 0 | 0 | 0 | 6 | 5 | 3 | 2 | 9 | 8 |
| Number of surviving animals | | 55 | 27 | | 27 | | 34 | | 17 | |

Thirty-three rats that had no treatment but which were kept for the same time as the experimental animals constituted controls in which no lesions were found.

by invasion of the adjacent liver tissue (Fig. 1, 2). The cells forming these tumours differed in some features from normal liver cells. A common type consisted of large cells with pale staining vacuolated cytoplasm (Fig. 1); another type consisted of deeper staining rather less differentiated cells (Fig. 2) but there were variations of all types between these. The tumours varied from 5 mm to 3 cm in diameter. In no rat with these tumours was a metastatic deposit seen.

*Cholangiomata* were typical tumours composed of proliferating cells with the recognizable morphological characteristics of bile duct cells, well differentiated into gland-like spaces between a small amount of connective tissue stroma. These tumours varied from 3 to 10 mm in diameter. No metastatic deposits were seen. *Cysts* were usually unilocular and lined by epithelium resembling that in the bile ducts.

On microscopic scanning of liver sections, localized collections of liver cells from 0·3 to 2·0 mm diameter were observed scattered randomly through the tissue. The cytology of the cells forming these lesions differed from normal. The common type was composed of pale cells with finely vacuolated cytoplasm (Fig. 3, 4) that resembled the cells in the common type of tumour (Fig. 1). A second type was composed of darker staining cells (Fig. 5), and others were composed of cells of intermediate type. An increased number of cells with pyknotic nuclei was seen in these lesions and mitotic figures were occasionally present, suggesting an increased rate of cell turnover. These groups of cells had enlarged by expansion since the surrounding liver cells were often thrust aside (Fig. 3, 4). Usually there was no evidence of invasive growth. The larger of these lesions (*e.g.* Fig. 4) resembled small hepatocellular tumours. In Table I they are classified as " focal proliferations ". Smaller and less clearly defined groups of similar cells were also frequent, probably of the same nature, and classified as " small focal proliferations ".

A further small group of lesions was also seen in the liver. These consisted of small groups of irregularly proliferated bile ducts (Fig. 6).

FIG. 1.—Photomicrograph of section of hepatocellular tumour 2 cm diameter showing invasive manner of growth, thrusting aside of adjacent liver due to expansive growth, and cell characteristics of a common type of tumour. H. and E. ×200.

FIG. 2.—Photomicrograph of section of hepatocellular tumour 1 cm diameter showing invasive growth, thrusting aside of liver cells due to expansive growth, and cell characteristics of another type of tumour. H. and E. ×200.

FIG. 3.—Photomicrograph of section of a focal proliferation of liver cells showing cytological characteristics. There is evidence of expansive growth. H. and E. ×120.

FIG. 4.—Photomicrograph of section of a larger focal proliferation of liver cells showing cell characteristics. There is clear evidence of expansive growth and a suggestion of invasive growth at one point. The section embraces the whole diameter of the lesion. Note similarity of cell type to that in the lesion in Fig. 3 and in the hepatocellular tumour in Fig. 1. H. and E. ×120.

FIG. 5.—Photomicrograph of section of a focal proliferation of a different cytological type. The cells are deeper staining and show probable invasion of the surrounding liver. Compare with the section of the hepatocellular tumour in Fig. 2. H. and E. × 120.

FIG. 6.—Photomicrograph of section of a " duct proliferation " of average size, showing the small area of atypical proliferation of bile ducts. H. and E. × 120.

*Lesions in the kidneys*

The kidney tumours were similar to those reported by other workers after administration of DMN and were classified as either " adenocarcinomata " or " nephroblastoma " (Magee and Barnes, 1962). However, some of the tumours in the first category appeared to be of a mixed type (Riopelle and Jasmin, 1969; Hard and Butler, 1970).

*Distribution of lesions in the liver and kidneys*

The numbers of lesions in the liver and kidneys 12 months after treatment with DMN are set out in Table I. Tumours were counted on naked eye examination and confirmed microscopically. Other lesions were counted by microscopic examination of sections of liver about 2 cm$^2$. The number of lesions seen in sections 5 $\mu$m thick is regarded as a measurable parameter of the incidence in the liver. Statistical data are presented in Table II.

DMN alone did not produce any kidney tumours in 27 surviving rats, but a small number of tumours and other lesions were found in the liver. When the DMN was given 42 hours after $CCl_4$ there was a significant yield of kidney tumours. The small increase in number of hepatocellular tumours is not significant but there is an increase in the number of focal proliferations. When DMN was given 60 hours after $CCl_4$ the number of kidney tumours was less than in the 42-hour group, but the yield of all types of lesion in the liver was increased.

When DMN (40 mg/kg) was given 42 hours after $CCl_4$ the yield of kidney tumours was much greater than with the dose of 20 mg/kg. The yield of hepatocellular tumours was not increased although the yield of focal proliferations was greater. On the other hand, the yield of cholangiomata and related types of duct lesions was increased.

There appears to be a correlation between the number of hepatocellular tumours with the number of " focal proliferations ". A similar relationship appears to be likely between the incidence of cholangiomata and the " duct proliferations ".

### DISCUSSION

After a dose of 2·5 ml/kg of $CCl_4$ the level of DMN-demethylase activity in the liver, upon which the production of the active intermediate responsible for the cytotoxic effects of DMN appears to depend, was greatly reduced for a period lasting between 42 and 72 hours, but the activity of this enzyme in the kidney was not changed (Pound *et al.*, 1973). The toxic effects of DMN were reduced during this period and began to return to normal levels after 60 hours.

The tumour-enhancing effect of $CCl_4$ on the kidneys may therefore be explained as a dose effect, that is, the diminished rate of metabolism of DMN 42 hours after $CCl_4$ ensured that more DMN was available to the kidneys and for a longer time so that more tumours were produced. After an interval of 60 hours, when the

TABLE II.—*Statistical Data on Incidence of Lesions in Table I*

| | | |
|---|---|---|
| DMN 42 hours after $CCl_4$ *vs* DMN alone | Heptaocellular tumours | Increase N.S. |
| | Focal proliferations (liver) | Increase $\chi^2 = 4{\cdot}5$, 1 d.f., $P < 0{\cdot}05$ |
| | Kidney tumours | Increase $\chi^2 = 7{\cdot}3$, 1 d.f., $P < 0{\cdot}01$ |
| DMN 60 hours after $CCl_4$ *vs* DMN 42 hours after $CCl_4$ | Hepatocellular tumours | Increase $\chi^2 = 2{\cdot}3$, 1 d.f., $0{\cdot}2 > P > 0{\cdot}1$ |
| | Focal proliferations (liver) | Increase $\chi^2 = 1{\cdot}4$, 1 d.f., N.S. |
| | Kidney tumours | Decrease $\chi^2 = 3{\cdot}63$, 1 d.f., $0{\cdot}1 > P > 0{\cdot}05$ |

metabolism of DMN by the liver was returning to normal, the tumour yield in the kidneys was reduced. The carcinogenicity of DMN for kidneys was strongly dose dependent (Riopelle and Jasmin, 1963). Rats fed a protein-free diet which lowered the rate of metabolism of DMN (Swann and McLean, 1971) can be given a single large dose of DMN with a large increase in the tumour yield (McLean and Magee, 1970; Hard and Butler, 1970). A similar increase of kidney tumours was obtained when a large dose of DMN (40 mg/kg) was possible under the protecting influence of $CCl_4$ given 42 hours earlier.

The situation regarding the liver is more complicated. This dose of $CCl_4$ (2·5 ml/kg) produced necrosis involving the inner one-third of the liver lobules, followed after a period of 24 hours by a phase of active DNA synthesis which reached a peak at 42 hours and a second peak at 60 hours. Mitotic activity was at a maximum at about 48 hours (Pound *et al.*, 1973). Thus the DMN was given at times of maximum DNA synthesis. Although the diminished rate of metabolism of DMN at 42 hours would impose a similar dose effect on the liver as on the kidney, in the liver (unlike in the kidney) the DMN-demethylase level was depressed. There is therefore an ambiguity that makes it difficult to correlate the tumour yields with any particular phase of cell replication. Nonetheless, the results are consistent with the view that DMN is a more effective carcinogen when reacting with cells synthesizing DNA than with cells during mitosis, because the tumour yield was increased at the 42-hour interval before the peak of mitotic activity, at a stage when DMN demethylase was depressed and presumably the active intermediate less available. The even greater tumour yield at the 60-hour interval occurred at a stage when active DNA synthesis was combined with levels of DMN-demethylase returning to normal. When a larger dose of DMN (40 mg/kg) was given 42 hours after $CCl_4$ the yield of hepatocellular tumours was not significantly increased although the yield of focal proliferations was. The production of hepatocellular tumours may not be strongly dose dependent. On the other hand, there was a significant increase in cholangiomata, duct proliferations and cysts, but the significance of these distributions needs further investigation.

The present results are of interest because 12 months after a dose of DMN, small groups of liver cells of different morphology and cytology to the surrounding normal liver cells were common. The expansive manner of growth and other characteristics of these lesions suggest that they are foci of liver cells proliferating independently and possibly arising from individual cells. It is significant that the number of these lesions in any experimental situation broadly correlated with the incidence of tumours. It seems possible that tumours may arise as a result of continued growth of these focal proliferations—that is, they are neoplasms *in embryo* as it were—or alternatively that they are susceptible to a further change leading to a neoplastic type of growth. It is not known if these focal proliferations may be seen soon after a single dose of DMN, nor is it known if they continue to grow, remain static in size after a time or finally regress.

Groups of cells with different morphology from the surrounding liver cells have been described in rats given a course of diethylnitrosamine (DEN) over some days, soon after the treatment and long before tumours appeared (Gössner and Friedrich-Freksa, 1964; Friedrich-Freksa, Gössner and Börner, 1969). These cells differed in enzyme constitution from normal liver cells and resembled those in the hepatocellular tumours found in animals that had the same treatment. This supported their thesis that tumours arise in or from these lesions. It was also found that the number of these lesions was increased when DEN was given after partial hepatectomy (Scherer and Hoffman, 1971). The descriptions and the illustrations of these lesions suggest that they are of the same nature as the focal proliferations in the present work.

This work was supported by grants from the Mayne Bequest Fund of the University of Queensland and the Queensland Cancer Trust. Lorraine Horn was supported by a National Health and Medical Research Council Undergraduate Research Scholarship.

### REFERENCES

Barnes, J. M. & Magee, P. N. (1954) Some Toxic Properties of Dimethylnitrosamine. *Br. J. ind. Med.*, **11**, 167.

Cameron, G. R. & Karunaratne, W. A. E. (1936) Carbon Tetrachloride Cirrhosis in Relation to Liver Regeneration. *J. Path. Bact.*, **42**, 1.

Chernozemski, I. N. & Warwick, G. P. (1970) Liver Regeneration and Induction of Hepatomas in B6AF$_1$ Mice by Urethan. *Cancer Res.*, **30**, 2685.

Clayson, D. B. (1962) *Chemical Carcinogenesis.* London: J. & A. Churchill.

Craddock, V. M. (1971) Liver Carcinomas Induced in Rats by Single Administration of Dimethylnitrosamine after Partial Hepatectomy. *J. natn. Cancer Inst.*, **47**, 899

Fiume, L., Campadelli-Fiume, G., Magee, P. N. & Holsman, J. (1970) Cellular Injury and Carcinogenesis. Inhibition of Metabolism of Dimethylnitrosamine by Aminoacetonitrile. *Biochem. J.*, **120**, 601.

Friedrich-Freksa, H., Gössner, W. & Börner, P. (1969) Histochemische Untersuchungen der Cancerogenese in der Rattenleber nach Dauergaben von Diäthylnitrosamin. *Z. Krebsforsch.*, **72**, 226.

Gössner, W. & Friedrich-Freksa, H. (1964) Histochemische Untersuchungen über die Glucose-6-phosphatase in der Rattenleber während der Kanzerisierung durch Nitrosamine. *Z. Naturf.*, **19b**, 862.

Hard, G. C. & Butler, W. H. (1970) Cellular Analysis of Renal Neoplasia: Light Microscopic Study of the Development of Interstitial Lesions Induced in the Rat Kidney by a Single Carcinogenic Dose of Dimethylnitrosamine. *Cancer Res.*, **30**, 2806.

Hoffman, J., Himes, M. B., Lapan, S., Riszki, R. & Post, J. (1955) Responses of the Liver to Injury: Effect of Acute Carbon Tetrachloride Poisoning. *Archs Path.*, **59**, 429.

Leevy, Carroll M., Hollister, R. M., Schmid, R., Macdonald, R. A. & Davidson, C. S. (1959) Liver Regeneration in Experimental Carbon Tetrachloride Intoxication. *Proc. Soc. exp. Biol. Med.*, **102**, 672.

McLean, A. E. M. & Magee, P. N. (1970) Increased Renal Carcinogenesis by Dimethylnitrosamine in Protein Deficient Rats. *Br. J. exp. Path.*, **51**, 587.

McLean, A. E. M. & Verschuuren, H. G. (1969) Effects of Diet and Microsomal Enzyme Induction on the Toxicity of Dimethylnitrosamine. *Br. J. exp. Path.*, **50**, 22.

Magee, P. N. & Barnes, J. M. (1962) Induction of Kidney Tumours in the Rat with Dimethylnitrosamine (N-nitrosodimethylamine). *J. Path. Bact.*, **84**, 19.

Magee, P. N. & Barnes, J. M. (1967) Carcinogenic Nitroso Compounds. *Adv. Cancer Res.*, **10**, 163.

Magour, S. & Nievel, J. G. (1971) Effect of Inducers of Drug Metabolizing Enzymes in Diethylnitrosamine Metabolism and Toxicity. *Biochem. J.*, **123**, 8p.

Mirvish, S. S. & Sidransky, H. (1971) Labelling *in vivo* of Rat Liver Proteins by Tritium-labelled Dimethylnitrosamine. Effect of Prior Treatment with 3-methylcholanthrene, Phenobarbitone, Dimethylformamide, Diethylformamide, Aminoacetonitrile, Ethionine and Carbon Tetrachloride. *Biochem. Pharmac.*, **20**, 3493.

Pound, A. W. (1968) Carcinogenesis and Cell Proliferation. *N.Z. med. J.*, **67**, 88.

Pound, A. W., Horn, L. & Lawson, T. A. (1973) Decreased Toxicity of Dimethylnitrosamine in Rats after Treatment with Carbon Tetrachloride. *Pathology.* In the press.

Riopelle, J. L. & Jasmin, G. (1963) Discussion sur la nature des Tumeurs Rénales Induites chez le Rat par la Dimethylnitrosamine. *Rev. Can. Biol.*, **22**, 365.

Riopelle, J. L. & Jasmin, G. (1969) Nature, Classification and Nomenclature of Kidney Tumours Induced in the Rat by Dimethylnitrosamine. *J. natn. Cancer Inst.*, **42**, 643.

Scherer, E. & Hoffmann, M. (1971) Probable Clonal Genesis of Cellular Islands Induced in Rat Liver by Diethylnitrosamine. *Eur. J. Cancer*, **7**, 369.

Schmähl, D., Krüger, F. W., Ivankovic, S. & Preissler, P. (1971) Verminderung der Toxizität von Dimethylnitrosamin bei Ratten und Mäusen nach Behandlung mit Disulfiram. *Arzneimittel-Forsch.*, **21**, 1560.

Somogyi, A., Conney, A. H., Kuntzman, R. & Solymos, B. (1972) Protection against Dimethylnitrosamine Toxicity by Pregnenolone-16α-carbonitrile. *Nature, New Biol.*, **237**, 61.

Swann, P. F. & McLean, A. E. M. (1971) Cellular Injury and Carcinogenesis: the Effect of a Protein-Free High-carbohydrate Diet on the Metabolism of Dimethylnitrosamine in the Rat. *Biochem. J.*, **124**, 283.

Venkatesan, N., Arcos, J. C. & Argus, M. F. (1968) Differential Effect of Polycyclic Hydrocarbons on the Demethylation of the Carcinogen Dimethylnitrosamine by Rat Tissues. *Life Sci.*, **7**, **1**, 1111.

Venkatesan, N., Argus, M. F. & Arcos, J. C. (1970) Mechanism of 3-methylcholanthrene-induced Inhibition of Dimethylnitrosamine Demethylase in Rat Liver. *Cancer Res.*, **30**, 2556.


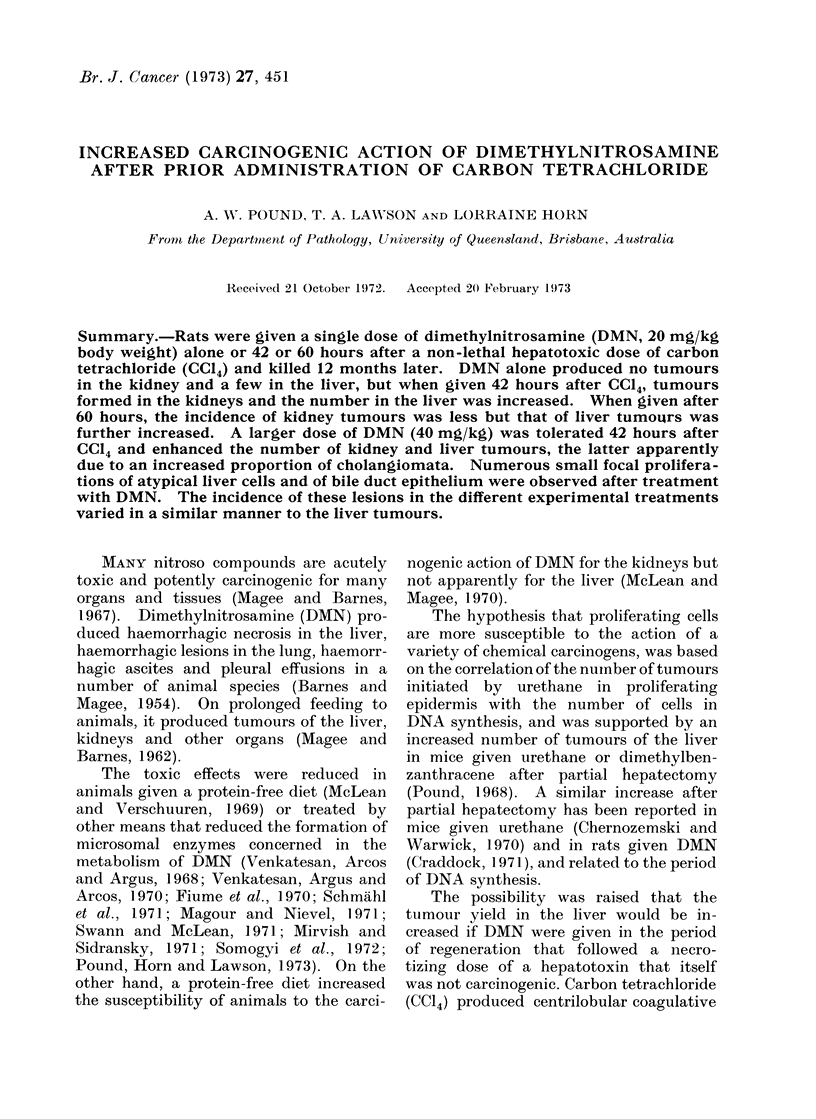

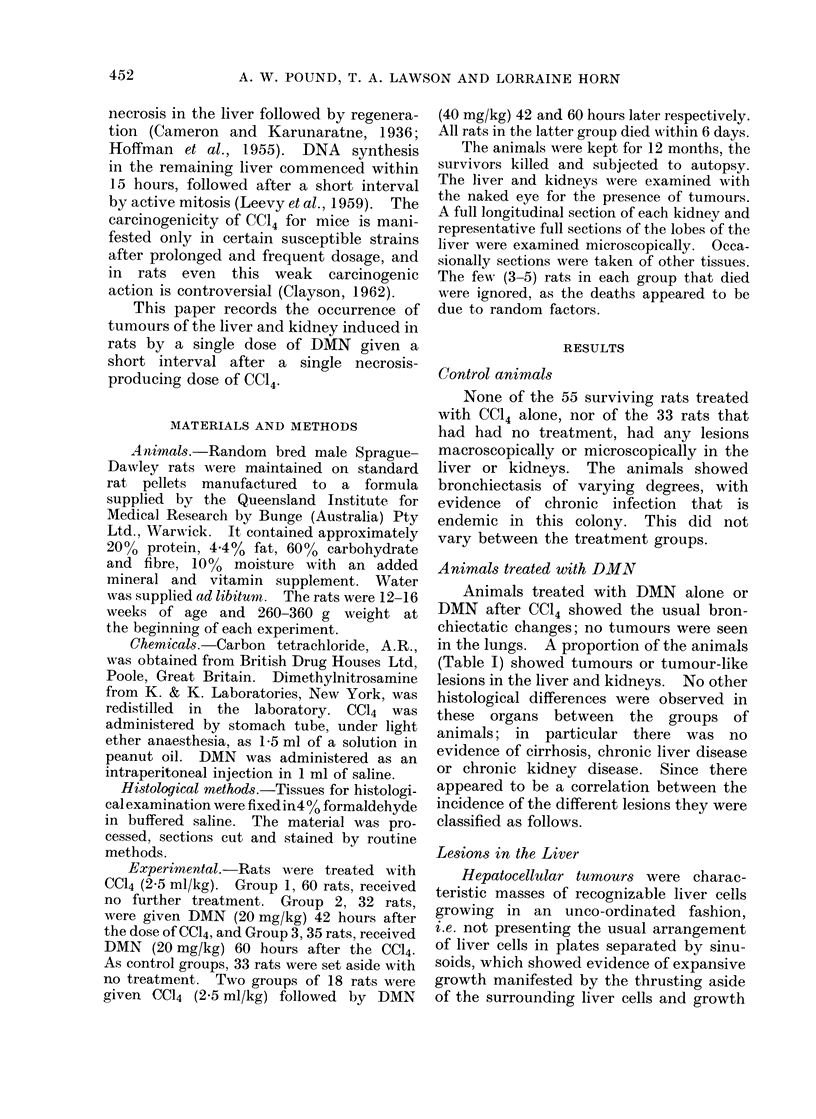

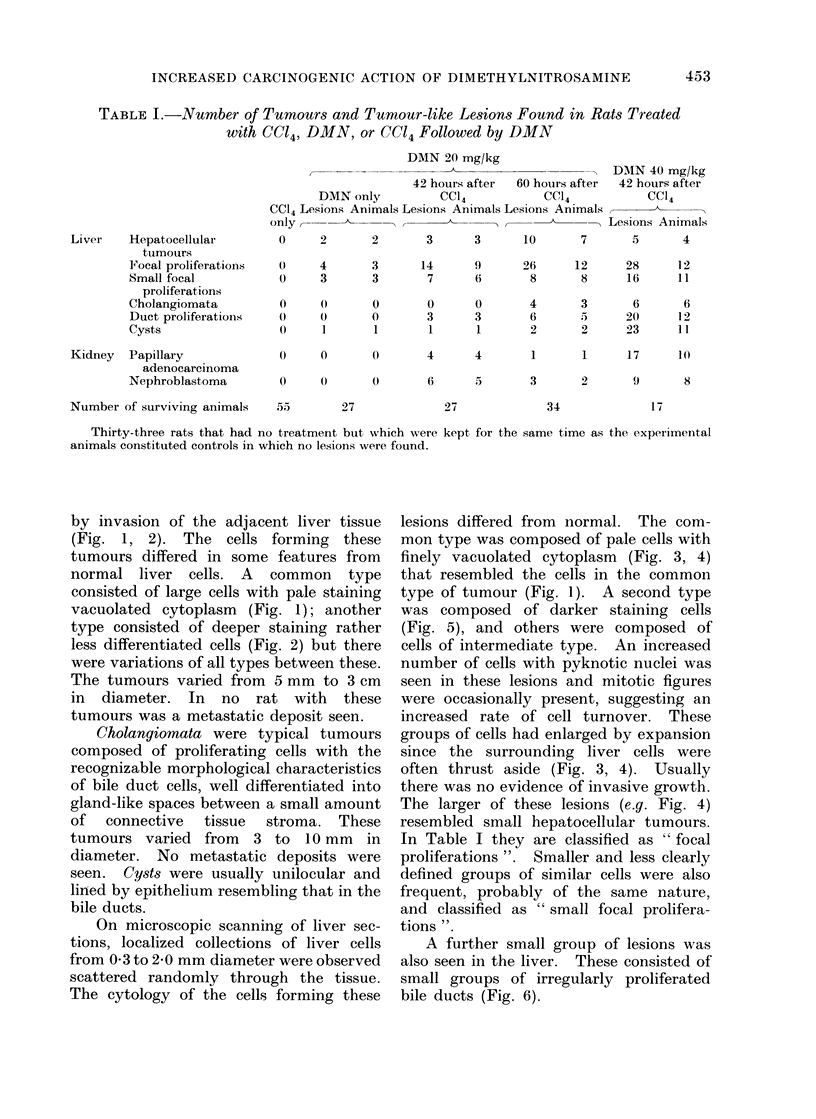

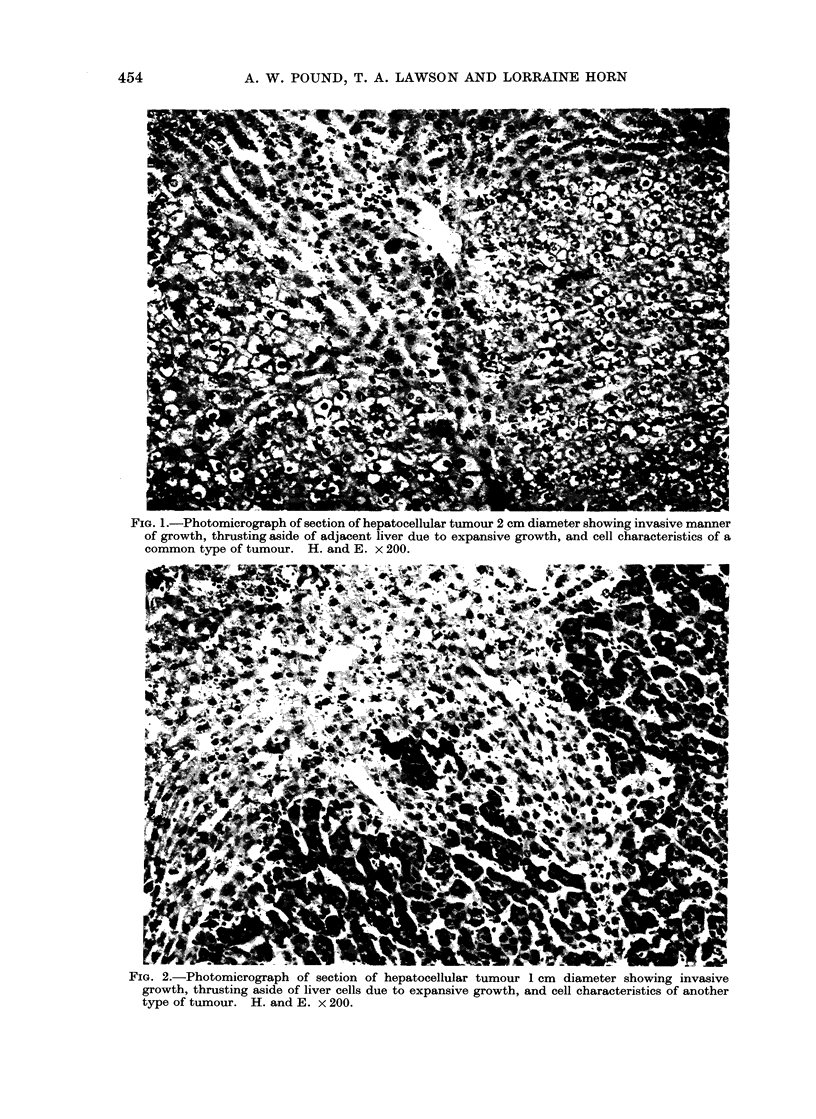

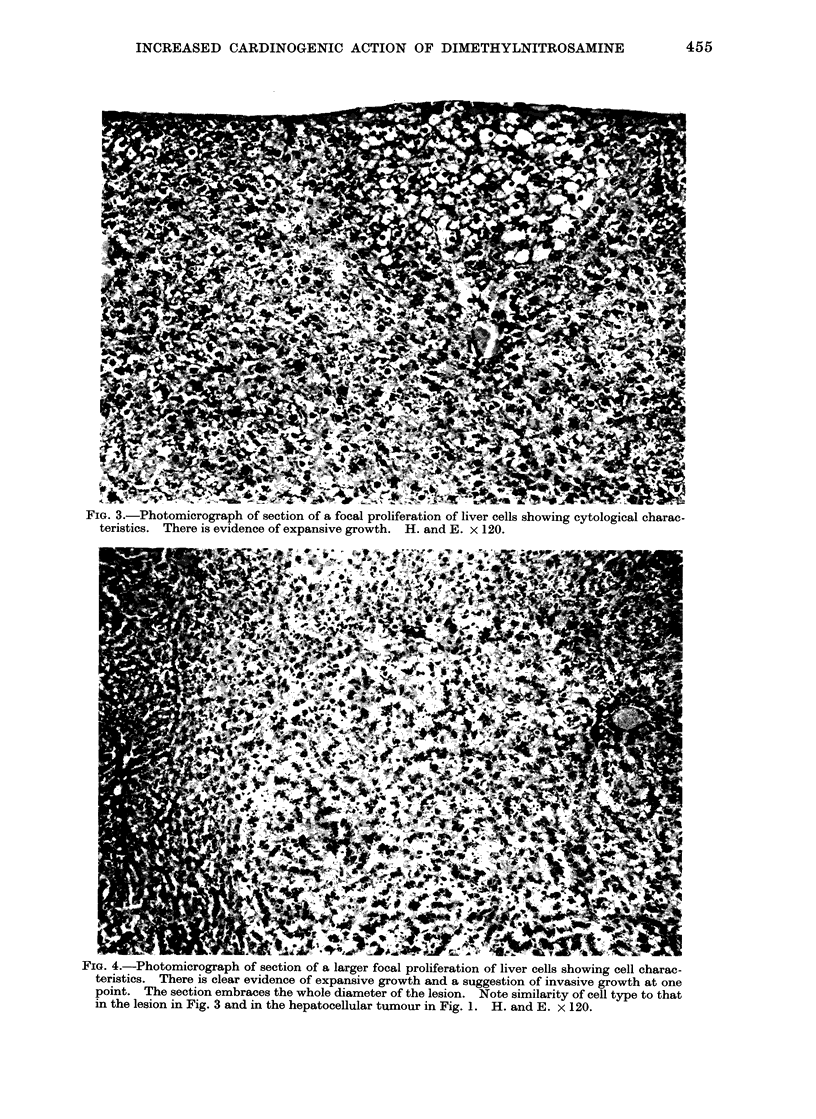

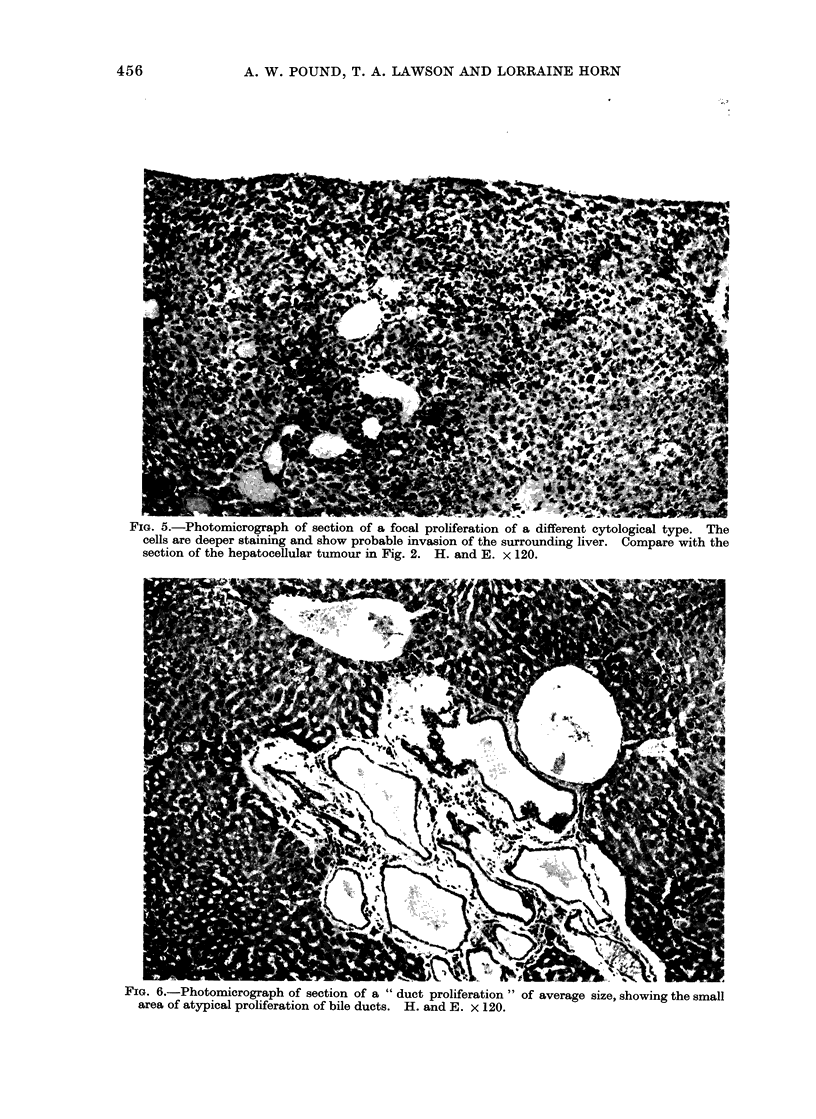

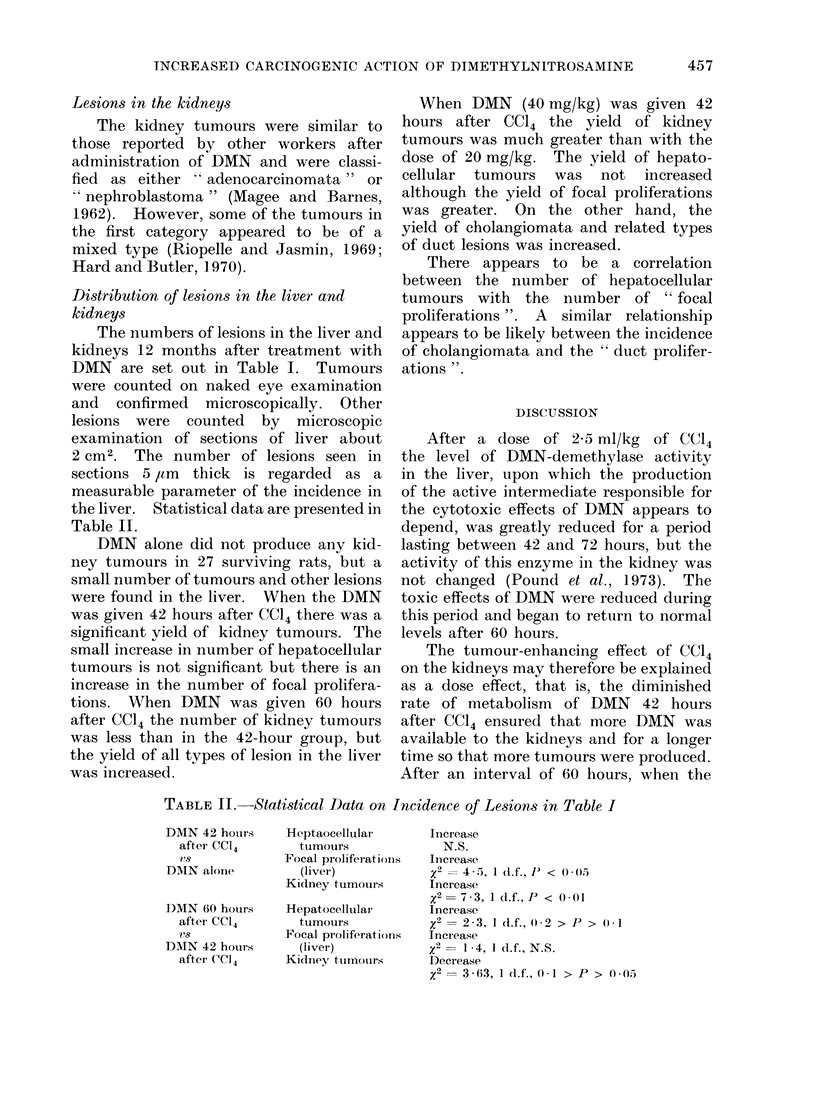

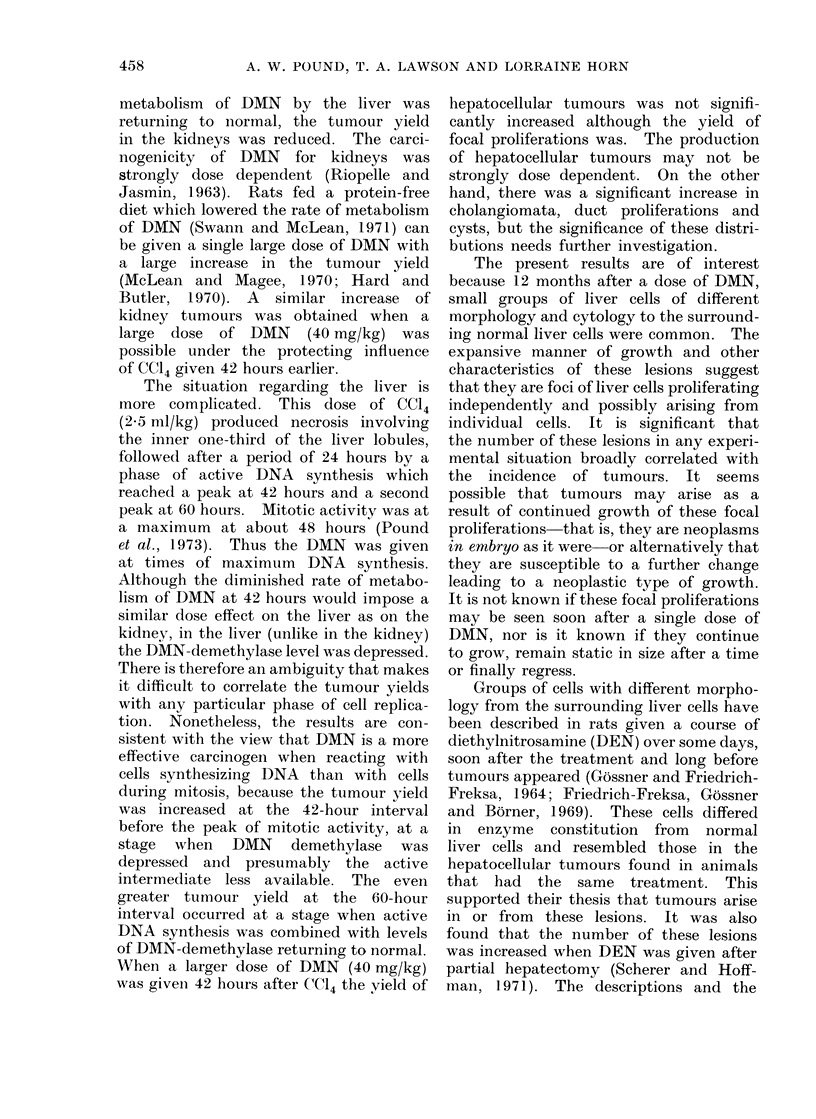

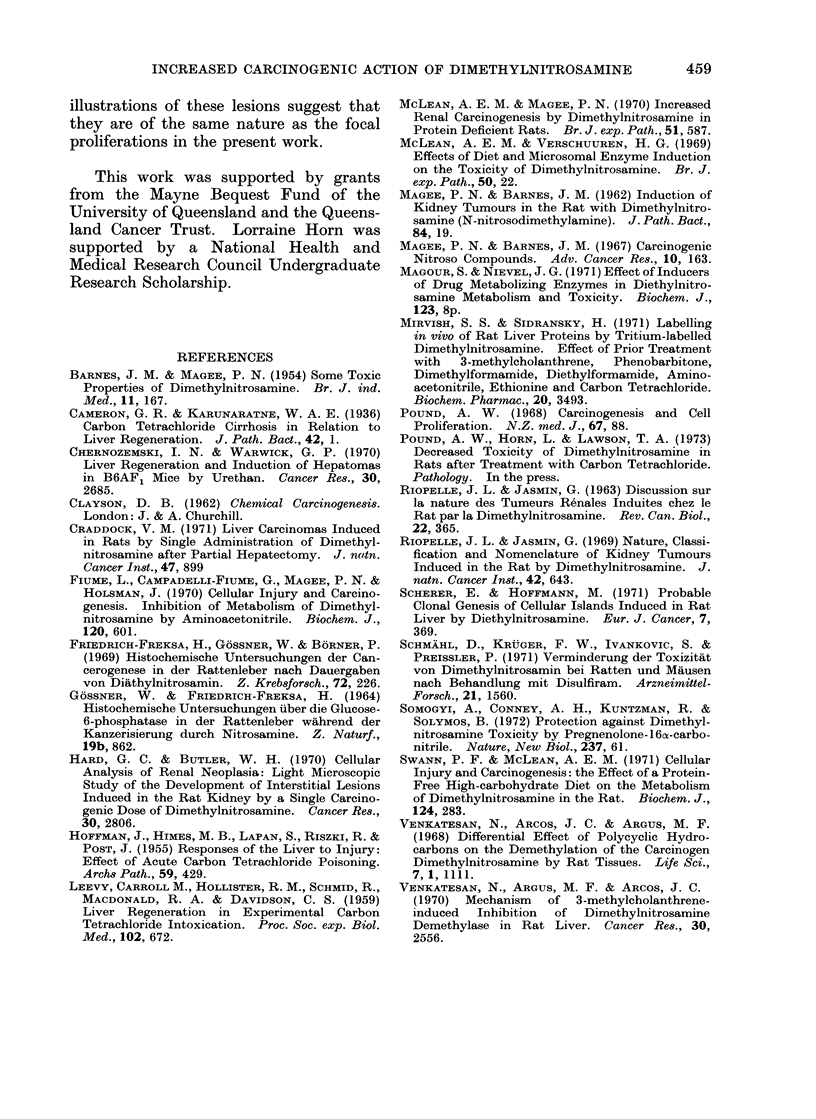


## References

[OCR_00677] BARNES J. M., MAGEE P. N. (1954). Some toxic properties of dimethylnitrosamine.. Br J Ind Med.

[OCR_00687] Chernozemski I. N., Warwick G. P. (1970). Liver regeneration and induction of hepatomas in B6AF mice by urethan.. Cancer Res.

[OCR_00697] Craddock V. M. (1971). Liver carcinomas induced in rats by single administration of dimethylnitrosamine after partial hepatectomy.. J Natl Cancer Inst.

[OCR_00703] Fiume L., Campadelli-Fiume G., Magee P. N., Holsman J. (1970). Cellular injury and carcinogenesis. Inhibition of metabolism of dimethylnitrosamine by aminoacetonitrile.. Biochem J.

[OCR_00710] Friedrich-Freksa H., Gössner W., Börner P. (1969). Histochemische Untersuchungen der Cancerogenese in der Rattenleber nach Dauergaben von Diäthylnitrosamin.. Z Krebsforsch.

[OCR_00730] HOFFMAN J., HIMES M. B., LAPAN S., RISZKI R., POST J. (1955). Responses of the liver to injury: effects of acute carbon tetrachloride poisoning.. AMA Arch Pathol.

[OCR_00722] Hard G. C., Butler W. H. (1970). Cellular analysis of renal neoplasia: light microscope study of the development of interstitial lesions induced in the rat kidney by a single carcinogenic dose of dimethylnitrosamine.. Cancer Res.

[OCR_00738] LEEVY C. M., HOLLISTER R. M., SCHMID R., MACDONALD R. A., DAVIDSON C. S. (1959). Liver regeneration in experimental carbon tetrachloride intoxication.. Proc Soc Exp Biol Med.

[OCR_00753] MAGEE P. N., BARNES J. M. (1962). Induction of kidney tumours in the rat with dimethylnitrosamine (N-nitrosodimethylamine).. J Pathol Bacteriol.

[OCR_00759] Magee P. N., Barnes J. M. (1967). Carcinogenic nitroso compounds.. Adv Cancer Res.

[OCR_00743] McLean A. E., Magee P. N. (1970). Increased renal carcinogenesis by dimethyl nitrosamine in protein deficient rats.. Br J Exp Pathol.

[OCR_00747] McLean A. E., Verschuuren H. G. (1969). Effects of diet and microsomal enzyme induction on the toxicity of dimethyl nitrosamine.. Br J Exp Pathol.

[OCR_00768] Mirvish S. S., Sidransky H. (1971). Labeling in vivo of rat liver proteins by tritium-labeled dimethylnitrosamine. Effect of prior treatment with 3-methylcholanthrene, phenobarbitone, dimethylformamide, diethyl-formamide, aminoacetonitrile, ethionine and carbon tetrachloride.. Biochem Pharmacol.

[OCR_00777] Pound A. W. (1968). Carcinogenesis and cell proliferation.. N Z Med J.

[OCR_00787] RIOPELLE J. L., JASMIN G. (1963). DISCUSSION SUR LA NATURE DES TUMEURS R'ENALES INDUITES CHEZ LE RAT PAR LE DIM'ETHYLNITROSAMINE.. Rev Can Biol.

[OCR_00793] Riopelle J. L., Jasmin G. (1969). Nature, classification, and nomenclature of kidney tumors induced in the rat by dimethylnitrosamine.. J Natl Cancer Inst.

[OCR_00799] Scherer E., Hoffmann M. (1971). Probable clonal genesis of cellular islands induced in rat liver by diethylnitrosamine.. Eur J Cancer.

[OCR_00805] Schmähl D., Krüger F. W., Ivankovic S., Preissler P. (1971). Vermindung der Toxizität von Dimethylnitrosamin bei Ratten und Mäusen nach Behandlung mit Disulfiram.. Arzneimittelforschung.

[OCR_00812] Somogyi A., Conney A. H., Kuntzman R., Solymoss B. (1972). Protection against dimethylnitrosamine toxicity by pregnenolone-16 -carbonitrile.. Nat New Biol.

[OCR_00818] Swann P. F., McLean A. E. (1971). Cellular injury and carcinogenesis. The effect of a protein-free high-carbohydrate diet on the metabolism of dimethylnitrosamine in the rat.. Biochem J.

[OCR_00825] Venkatesan N., Arcos J. C., Argus M. F. (1968). Differential effect of polycyclic hydrocarbons on the demethylation of the carcinogen dimethylnitrosamine by rat tissues.. Life Sci.

[OCR_00832] Venkatesan N., Argus M. F., Arcos J. C. (1970). Mechanism of 3-methylcholanthrene-induced inhibition of dimethylnitrosamine demethylase in rat liver.. Cancer Res.

